# Textbook Outcome after Gastrectomy for Gastric Cancer Is Associated with Improved Overall and Disease-Free Survival

**DOI:** 10.3390/jcm12165419

**Published:** 2023-08-21

**Authors:** Candan Çetinkaya-Hosgör, Philippa Seika, Jonas Raakow, Dino Kröll, Eva Maria Dobrindt, Max Magnus Maurer, Friederike Martin, Ramin Raul Ossami Saidy, Peter Thuss-Patience, Johann Pratschke, Matthias Biebl, Christian Denecke

**Affiliations:** 1 Chirurgische Klinik, Campus Charité Mitte, Campus Virchow-Klinikum, Charité Universitätsmedizin, 10117 Berlin, Germany; 2 Department of Surgery, Division of Surgical Sciences, Beth Israel Deaconess Medical Center, Harvard Medical School, Boston, MA 02215, USA; 3 Berlin Institute of Health, Charité Universitätsmedizin, 10117 Berlin, Germany; 4 Division of Transplant Surgery, Department of Surgery, Brigham and Women’s Hospital, Harvard Medical School, Boston, MA 02215, USA; 5 Medizinische Klinik mit Schwerpunkt Hämatologie, Onkologie und Tumorimmunologie, Campus Charité Mitte, Campus Virchow-Klinikum, Charité Universitätsmedizin, 10117 Berlin, Germany; 6 Department of General, Visceral, Thoracic and Transplant Surgery, Congregational Hospital Linz, Seilerstätte 4, 4010 Linz, Austria; 7 Kepler University Hospital Linz, Med. Campus III, Krankenhaussstrasse 7a, 4020 Linz, Austria

**Keywords:** gastric surgery, textbook outcome, gastric cancer, minimally invasive gastrectomy

## Abstract

(1) Background: The complexity of the perioperative outcome for patients with gastric cancer is not well reflected by single quality metrics. To study the effect of the surgical outcome on survival, we have evaluated the relationship between textbook outcome (TO)—a new composite parameter—and oncological outcome. (2) Methods: All patients undergoing total gastrectomy or trans-hiatal extended gastrectomy for gastric cancer with curative intent between 2017 and 2021 at our institution were included. TO was defined by negative resection margins (R0); collection of ≥25 lymph nodes; the absence of major perioperative complications (Clavien–Dindo ≥ 3); the absence of any reintervention; absence of unplanned ICU re-admission; length of hospital stay < 21 days; absence of 30-day readmission and 30-day mortality. We evaluated factors affecting TO by multivariate logistic regression. The correlation between TO and long-term survival was assessed using a multivariate cox proportional-hazards model. (3) Results: Of the patients included in this study, 52 (52.5 %) achieved all TO metrics. Open surgery (*p* = 0.010; OR 3.715, CI 1.334–10.351) and incomplete neoadjuvant chemotherapy (*p* = 0.020, OR 4.278, CI 1.176–15.553) were associated with failure to achieve TO on multivariate analysis. The achievement of TO significantly affected overall survival (*p* = 0.015). TO (*p* = 0.037, OD 0.448, CI 0.211–0.954) and CCI > 4 (*p* = 0.034, OR 2.844, CI 1.079–7.493) were significant factors affecting DFS upon univariate analysis. In multivariate analysis, CCI > 4 (*p* = 0.035, OR 2.605, CI 0.983–6.905) was significantly associated with DFS. (4) Conclusions: We identified patient- and procedure-related factors influencing TO. Importantly, achieving TO is strongly associated with improved long-term survival in gastric cancer patients and merits further focus on surgical quality improvement efforts.

## 1. Introduction

Gastric cancer (GC) remains one of the most common cancers worldwide and the annual burden of GC is predicted to increase to 1.8 million new cases and 1.3 million deaths by 2040 [1]. The treatment algorithm for gastroesophageal adenocarcinoma varies according to the extent of the disease but either involves a primarily surgical approach or a multimodal approach, including either neoadjuvant chemotherapy or chemoradiation followed by surgery [2].

A decrease in perioperative morbidity and mortality in recent years was attributed in part to the development and adoption of laparoscopic or robotic–assisted surgical approaches. However, postoperative morbidity and mortality remain challenging despite the above-mentioned techniques and improvements in perioperative management [3,4]. Postoperatively, Clavien–Dindo (CD) complications ≥ 3a, such as pneumonia and surgical site infection, in particular anastomotic leakage (AL), represent major challenges and are associated with a poor prognosis [5]. The rate of perioperative morbidity and mortality is also a risk factor for failure to complete Multimodal Treatment (MMT) or adjuvant chemotherapy [6]. The rate of perioperative morbidity and mortality varies across the literature with complications CD > 2 being reported in 5–43% of patients after gastrectomy [7,8]. Besides surgical complications, other metrics for surgical outcome include positive resection margins and adequate lymph node sampling. A number of recent studies have reported positive resection margins in 1.0% to 20.0% of patients [9] and inadequate lymph node sampling in 5.8% to 82.4% [10]. Due to the lack of a defined method for tracking these parameters, evaluations of their effects on oncological outcomes also vary widely, which makes quality improvement challenging [5,6].

Recently, efforts have been made to standardize and centralize gastric surgery and therefore improve surgical and oncological outcomes, with certification for centers based on outcome and volume of operations performed [11]. Increased experience in gastrectomy, both at the centre and individual level, have been associated with decrease in perioperative mortality [12]. However, a single variable such as morbidity or overall survival cannot reflect the multifaceted nature of surgical quality. Since its introduction in 2017, the concept of textbook outcome (TO) has garnered considerable attention, being initially described by the DUCA group as an embodiment of the “ideal” surgical outcome [13]. TO represents a more comprehensive overview of a patient’s hospitalization than individual quality indicators and some studies have proposed the use of TO as a parameter for surgical management [14,15]. Since its introduction, many novel TO classifications aimed at clinical and prognostic applications have been suggested. Despite the growing interest in the TO, it is essential to consider the limitations of its definition, as highlighted by the PRESTO group in their subsequent study [15] Careful parameter selection can facilitate reproducibility and the widespread adoption of this TO in clinical practice. Currently, no consistent definition for TO has been established as a standard in gastric surgery. The rate of TO, as well as patient- and procedure-related factors that may influence TO, remain unclear. In this study, we identify prognostic factors predicting TO at our institution, and assess the impact of achieving TO on overall survival (OS) and disease-free survival (DFS).

## 2. Patients and Methods

Patients undergoing total gastrectomy or trans-hiatal extended gastrectomy for primary gastric adenocarcinoma were included in this retrospective observational study from a prospectively kept institutional database. Exclusion parameters included: multi-visceral or atypical resection, metastatic disease, palliative intent, subtotal gastrectomy, and emergency surgery. For all patients, patient characteristics such as age, sex, Charlson Comorbidity Index (CCI) and the American Society of Anesthesiologists (ASA) score were determined. CCI is a weighted index that aims to predict the risk of death within 1 year of hospitalization for patients with specific comorbid conditions [16]. Patients were analyzed by CCI tertiles (CCI 0–4; CCI 5+). Additionally, a comprehensive histopathological examination of the resected GEJ samples was conducted. This assessment included evaluation of tumor subtype, grade, size, invasion depth, margins, lymph nodal involvement and inflammatory changes. Findings compatible with esophageal carcinoma were not detected through both preoperative endoscopic evaluations and histopathological analysis of the resected GEJ samples. Moreover, the applied chemotherapy regimen as well as relevant perioperative laboratory parameters were extracted from the database. The patients were stratified into two groups according to the achievement of TO during the postoperative course.

### 2.1. Data Collection

**Figure 1:**
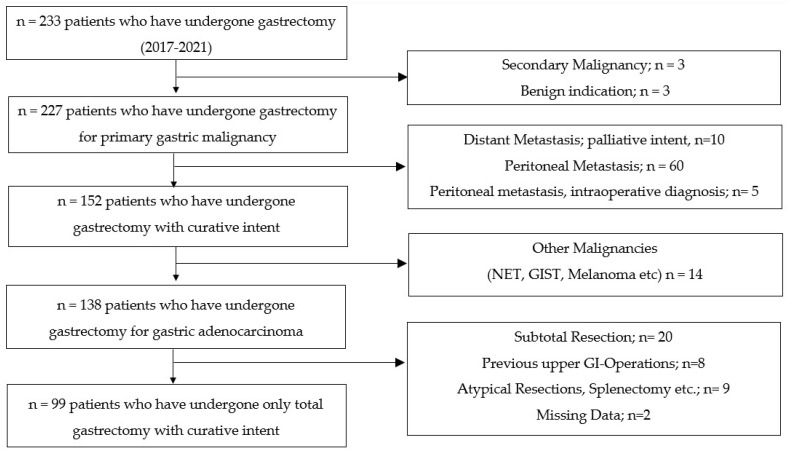
Inclusion and exclusion criteria.

Between January 2017 and December 2021, 233 patients underwent total, subtotal or trans-hiatal extended gastrectomy or atypical gastric resection at our institution (Figure 1). From a total of 233, 227 patients underwent gastrectomy for primary gastric malignancy. Among these 227 patients, 152 underwent gastrectomy with curative intent. Among 152 patients, 138 had adenocarcinoma while the remainder was excluded due to various other forms of GC (4 Neuroendocrine tumors (NET), 7 gastrointestinal stromal tumors (GIST), 1 malignant melanoma, 1 multiple endocrine neoplasia (MEN) and 1 synovial sarcoma). 37 patients were excluded because of multi-visceral resections, subtotal gastrectomy, and previous gastrointestinal resections. Finally, 99 patients after gastrectomy for primary gastric adenocarcinoma with curative intent were included after the other 2 patients were excluded because of missing data. At the time of resection, there was no evidence of metastatic disease in any of these patients.

### 2.2. Preoperative Evaluation and Multimodal Therapy

All patients underwent routine preoperative evaluation, including Esophagogastroduodenoscopy (EGD) and staging laparoscopy to exclude peritoneal metastasis and were discussed by multidisciplinary tumor board. Treatment for GC was recommended based on tumor board decision according to the German S3 guideline [17]. MMT is considered standard for patients with positive lymph nodes in perioperative staging or tumor stages >T2 at our institution. Unless otherwise noted, all tumor stages in this research correspond to postoperative histological classifications. After discharge from hospital, surgical outpatient follow-up was performed after 7 days. All cases were reevaluated postoperatively by the multidisciplinary tumor board. If neoadjuvant chemotherapy was recommended, this was initiated 6 weeks postoperatively.

### 2.3. Surgical Procedure

Open resection is performed via median laparotomy, with an incision that extends from the xiphoid to the umbilicus.

In laparoscopic surgery (LS), the abdominal cavity is accessed by a muscle-sparing transrectal incision in the left mid-abdomen and the introduction of a hand port (GelPort, Applied Medical, Rancho Santa Margarita, CA, USA). Following the establishment of pneumoperitoneum, the situs is explored. Following oncological resection and D2-lymphadenectomy, a linear stapler is used to transect the duodenum and esophagus, and Roux-en-Y esophagojejunostomy is performed for reconstruction. To assure tumor-free resection margins, intraoperative pathologic assessment is conducted on all patients by frozen section inspection. In the event of tumor infiltration, further resection is conducted until frozen section evaluation confirms tumor-free resection margins. In particular, the jejunojejunostomy and preparation of the alimentary limb are conducted in an open manner, while the esophagojejunostomy is performed manually end to side as a 25mm or 29mm circular anastomosis inserted in the jejunum. Circular anastomosis is performed utilizing either an Echelon circular^TM^ powered stapler (Johnson & Johnson Medical GmbH, Ethicon, Irvine, CA, USA) or the DST Series™ EEA™ OrVil™ Device (Medtronic, Minneapolis, MN, USA) introduced on the previously made purse-string into the esophagus. Linear anastomosis is performed with a 60 mm linear stapler (Echelon^TM^+ stapler, Johnson & Johnson Medical GmbH, Ethicon, Irvine, CA, USA), and the posterior wall of the esophagus and the anti-mesenteric side of the jejunum are anastomosed side by side with the overlap method [18]. Intraoperative endoscopy and air leak tests are used to confirm the anastomosis’s integrity. Before the abdominal cavity is closed, a perianastomotic drain is inserted. Trans-hiatal extended gastrectomy is performed as previously described [11] with the same anastomotic techniques described above.

### 2.4. Perioperative Management

Postoperatively, all patients were admitted to a surgical Intensive Care Unit. After beginning oral fluid intake on the first postoperative day (POD) and beginning early physical mobility on the first POD, progressive enteral food intake was initiated on the third POD if peristalsis was not affected. If the outflow was less than 150 mL/24h, perianastomotic drains were removed.

### 2.5. Histological Evaluation

Histopathological evaluation was performed according to a standardized gastrointestinal (GI) histopathology protocol. Esophageal and gastric specimens were examined including assessment of the resection margins and lymph nodes according to the tumor node metastasis (TNM) staging system. All cases prior to 2016 were restaged after the implementation of the eighth edition of the TNM classification in January 2016 [19]. Partial signet ring cell carcinoma (SRCC) was defined as having an SRCC component of ≤50%.

### 2.6. Definition of Textbook Outcome

All perioperative and postoperative complications during the perioperative course were prospectively collected in the database and classed according to the classification proposed by Clavien et al. (Clavien Dindo Classification, CD) [20]. Textbook outcome definition was adapted from Levy et. al. [12] and defined as (a) negative resection margin (R0), (b) 25 or more lymph nodes sampled, (c) no postoperative complication CD ≥ 3, (d) no re-interventions, (e) no unplanned ICU/IMC admission, (f) length of stay less than 21 days, (g) no readmission within 30-days or less of discharge and (h) no 30-day mortality following surgery.

### 2.7. Statistical Analysis

Statistical analysis was performed using JMP^®^ Pro, Version 16.0.0. Student’s *t*-test or ANOVA for continuous variables and Chi-squared test for categorical variables were used to compare demographic data between the two groups. Multivariate analysis was performed using logistic regression modelling. Survival analysis was performed by Kaplan Meier analysis and 1 and 2-year survival rates were reported. The association between the achievement of TO metrics and long-term survival was determined by multivariable Cox proportional-hazards model. A *p*-value less than 0.05 was considered significant.

## 3. Results

### 3.1. Reasons for Failure to Achieve Textbook Outcome

Of 99 patients included in the study, 52 (52.5%) achieved all TO metrics. Failure to achieve textbook outcome was most commonly a result of the occurrence of postoperative complications (CD ≥ 3) (19.2%) followed by the need for reinterventions (17.2%). Perioperative reinterventions included Esophagogastroduodenoscopy (EGD) in 10.1% of patients and CT-guided drainage of intraabdominal fluid collection in 14.1% of cases. In the case of perioperative complications (CD ≥ 3), 11.11% were of pulmonary origin, followed by infectious (8.1%) complications and the rate of AL (6.1%.). Two patients (1.8%) died during the first 30-days after surgery (Table 1).

### 3.2. Baseline Characteristics

The patient characteristics and perioperative outcomes are shown in Table 2. No differences in terms of BMI (25.06 vs. 26.34, *p* = 0.195) or pre-operative anemia (*n* = 38 (74.5%) vs. *n* = 29 (63.0%), *p* = 0.222) were seen between the groups. Furthermore, no differences were seen between male and female patients (*p* = 0.987), the patients with higher ASA score or lower ASA score (*p* = 0.185) and concomitant comorbidities (CCI ≥ 5, *n* = 24 (46.2%) vs. *n* = 30 (63.8%), *p* = 0.078). In contrast, the patients in the Non-TO group were significantly older (61.27 vs. 67.04, *p* = 0.032), and more frequently suffered from hyperlipidemia (*n* = 9 (17.3%) vs. *n* = 18 (38.3%), *p* = 0.019).

### 3.3. Tumor Location and Histopathological Characteristics

The location of the tumors and the distribution of all histopathological subtypes did not vary between the groups (Table 3). Importantly, nodal stages as well as perineural invasion, venous invasion and lymphatic vessel invasion were equally distributed.

### 3.4. Multimodal Therapy and Perioperative Characteristics

There was a significant difference in the completion of neoadjuvant therapy between the two groups. More patients received neoadjuvant chemotherapy (NCT) in the TO group (*n* = 50 (96.2%) vs. *n* = 36 (76.6%), *p* = 0.004). While this may be attributed to the trend towards advanced tumor stage in this group, NCT was also completed more consistently (*n* = 46 (88.5%) vs. *n* = 34 (72.3%), *p* = 0.042). This may be attributed to the significantly lower age seen in this group. The chemotherapy regimen consisted of FLOT (Fluorouracil, Leucovorin, Oxaliplatin and Docetaxel) in 78.8% (*n* = 41) of patients in the TO group and 55.3% (*n* = 26) in the non-TO group (*p* = 0.019). Finally, while adjuvant therapy was also recommended and administered at a higher rate in the TO group with FLOT (55.6% (*n* = 15) vs. 31.0% (*n* = 9)), many patients in the non-TO group (44.8% (*n* = 13) vs. 14.8% (*n* = 4)) did not receive adjuvant chemotherapy (*p* = 0.077). However, this trend was not significant. Interestingly, amongst patients who begun treatment with adjuvant chemotherapy, this was discontinued significantly more often in the non-TO group (TO *n* = 11 (40.7%) vs. Non-TO n = 16 (59.3%), *p* = 0.049). The non-completion of adjuvant therapy can serve as a surrogate parameter for individuals with more pre-existing conditions. However, if patients are unable to complete this additional therapy, it could also indicate a higher burden of complications related to the surgical procedure. This would imply that the decision not to pursue adjuvant therapy is not a deliberate choice but rather a consequence of the deterioration of patient’s overall health condition due to surgical complications. While this may also be due to the age and general constituency of the patient group, the achievement of TO certainly contributes to a successful progression to, and completion of, adjuvant therapy.

There was no significant difference in terms of the type of resection (total gastrectomy; TO *n* = 36 (69.2%) vs. Non-TO *n* = 26 (55.3%), *p* = 0.153) between the two groups. While THE gastrectomy is considered a more technically challenging procedure, it did not negatively affect the achievement of TO in this study. The surgical modality also varied, with more open resections being performed in the non-TO group (*n* = 8 (15.4%) vs. *n* = 19 (40.4%), *p* = 0.004). Similarly, a significantly longer length of hospital stay was seen in the non-TO group (TO, 12.67 days vs. non-TO, 22.28 days; *p* = <0.001). While this may result from the higher rate of complications in this group, length of hospital stay may also, independently result in non-TO (Table 4).

### 3.5. Factors Affecting Textbook Outcome

Significant factors affecting textbook outcome include age (*p* = 0.029; OR 6.997, CI 1.133–43.212), Open Surgery (*p* = 0.004; OR 3.894, CI 1.528–10.699) and non-completion of NCT (*p* = 0.002; OR 7.638, CI 1.595–36.584). Upon multivariate analysis, significant parameters affecting the achievement of TO remained open Surgery (*p* = 0.010; OR 3.715, CI 1.334–10.351) and completion of NCT (*p* = 0.020; OR 4.278, CI 1.176–15.553). Discontinuation of neoadjuvant chemotherapy (NCT) due to intolerance reflects the advanced age, and a trend towards higher ASA and CCI scores is also seen in this group (Table 5).

### 3.6. Textbook Outcome and Survival Outcome

**Figure 2:**
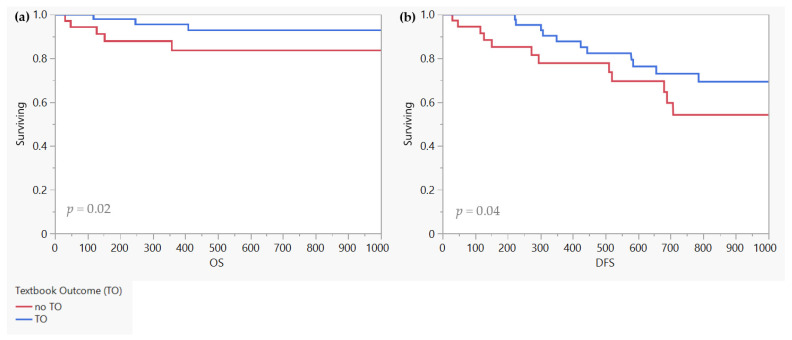
OS (**a**) and DFS (**b**). All patients with 30-day mortality or failure to follow up after 30 days postoperatively were excluded in both (**a**,**b**). Total patients included *n* = 86, non-TO = 34, TO = 52.

In survival analysis, we excluded 13 patients with either a perioperative death within 30 days or a follow up time of under 30 days. All remaining patients were included (*n* = 86, non-TO = 34, TO = 52). As mortality within 30 postoperative days is one of the criteria for TO, inclusion of these cases introduces a confounding factor into the survival analysis. Furthermore, it is important to note that the 30 day mortality rate pertains specifically to the perioperative period. The focus of this analysis, however, lies on the evaluation of the recurrence rate and medium to long-term survival outcomes. The mean follow up time was 657 days (8–2010 days). The disease-free survival rate at 1 year was 77.9% (non-TO) and 87.7% (TO), at two years 54.2% (non TO) and 69.4% (TO) (DFS, *p* = 0.0328) in this cohort. The rate of overall survival after 1 year also varied significantly between the two groups (non-TO 84.9% vs. TO 97.5%, *p* = 0.024) (Figure 2).

TO (*p* = 0.037, OD 0.448, CI 0.211–0.954), and CCI > 4 (*p* = 0.034, OR 2.844, CI 1.079–7.493) were significant factors affecting DFS upon univariate analysis (Table 6). In multivariate analysis, CCI > 4 (*p* = 0.035, OR 2.605, CI 0.983–6.905) was significantly associated with DFS.

## 4. Discussion

In recent years, the concept of the TO as a quality metric depicting the ideal surgical outcome has been used in complex oncological surgery. TO is a composite parameter that comprehensively represents the ideal postoperative course.

A number of recently published studies aim to define TO. The most common classification guidelines were published by the Presto [15] and DUCA [13] working groups. Other studies have modified these parameters to suit individual operations or operative subspecialties. According to the Population Registry of Esophageal and Stomach Tumors of Ontario (PRESTO) group, TO was defined by: negative resection margins; collection of >15 lymph nodes; the absence of perioperative complications (CD *≥* 3); the absence of reinterventions; absence of unplanned ICU re-admission; length of stay 21 days; absence of 30-day readmission and 30-day mortality; [15]. The inclusion of alternate parameters in the classification of TO has been proposed by some authors. “Textbook outcome” in the DUCA includes all the aforementioned parameters, as well as a radical resection according to the surgeon at the end of the operation and no intraoperative complications (CD 1–5). Sedlak et al. suggest that perioperative chemotherapy compliance should be included, as this reflects TO of the MMT rather than surgery alone [21]. The authors suggest that completion of NCT should be included in the classification. We did not include this in our definition as our intention was to assess the effect of optimal surgical outcome, as an independent parameter, on OS. Through the exclusion of this parameter in the definition, we were able to show that achievement of TO can help the patient achieve adjuvant chemotherapy contributing to the improved OS. In the present study, TO was defined as proposed by Levy et al. [15]. However, we considered a perioperative LN resection of 25 lymph nodes instead of 15 lymph nodes. According to the Japanese GC Treatment Guidelines, a minimum of 16 lymph nodes should be dissected for early-stage GC, while a minimum of 25 lymph nodes should be dissected for advanced-stage GC to improve the accuracy of staging and reduce the risk of under-staging. Several studies have reported that more extensive lymph node dissection (including more than 25 lymph nodes) can improve survival in patients with advanced GC [22].

In the present study, we saw a TO rate of 59.5%. Chen et al. reported a similar rate of TO at 56.5% [23]. However, this is higher than many of the TO rates reported in the literature, despite the use of a stricter definition of TO, with a minimum of 25 lymph nodes resected instead of the previously recommended 15. In a study with 1892 GC patients, Levy et al. showed a 22% achievement of all TO metrics in patients after gastrectomy for GC [15]. One possible reason for the differing rate of TO in our study may be attributed to the experience and case numbers of the senior consultants performing the operations. Laparoscopic surgery is associated with a longer learning curve than open surgery. Higher case volume can impact certain quality indicators included in textbook outcome metrics accounting. However, the learning curve had been completed by the beginning of this study, whilst other studies may have not taken this parameter into account [11]. Another possible reason may be the implementation of enhanced recovery after surgery (ERAS) during perioperative care, which may have contributed to better outcomes. Additionally, our study may have had a more homogeneous patient population, with fewer comorbidities and better overall health, which could have contributed to the higher TO rate observed. When it comes to the definition of postoperative mortality, studies also differ. In our study, we included the 30-day mortality, adapted from Levy et al. However, defining postoperative mortality as 30-day mortality may lead to an underestimation of the actual perioperative mortality, which may account for the higher rate of TO seen in our study.

Our study revealed that age, open surgery, hyperlipidemia and non-completion of NCT were significant factors affecting the achievement of TO in patients who underwent gastrectomy for GC. On multivariate analysis, we found open surgery to be a significant factor influencing the incidence of TO achievement. The use of open surgery was found to have a negative impact on the achievement of textbook outcome, with a higher likelihood of adverse events and longer hospital stay. It is commonly assumed that open surgery for GC is only necessary for patients with a high ASA or CCI score or a large tumor, and thus may be a surrogate parameter for these factors when considering TO. However, tumor size, ASA score and CCI did not differ significantly between the groups, although a trend towards higher scores was notable in the non-TO group. This suggests that there was no bias in patient selection in the laparoscopic surgery group. These data indicate that open surgery is not merely a surrogate parameter but is independently associated with failure to achieve TO. This is in line with other studies in the literature. Chen et. al. reported non-lower tumors, open surgery, and > 200 mL blood loss were independent risk factors for non-TO patients on multivariate logistic regression [23]. Similarly, Bolger et al. corroborated these findings reporting that a minimal invasive approach is associated with achievement of TO in both gastric and esophageal surgery [24]. In agreement, a recent metanalysis revealed a reduced 3-year mortality for patients undergoing laparoscopic surgery [25]. This suggests that age and comorbidity influence TO to a lesser extent, and there is a clear benefit of laparoscopic surgery wherever possible in terms of TO.

The results emphasize the importance of establishing minimal invasive surgical approach in achieving favorable outcomes in GC surgery. In cases where open surgery is deemed necessary, efforts should be made to optimize care trajectories to improve patient outcomes. Of note, there were more patients with GEJ tumors and trans-hiatal extended gastrectomy (THE) in the non/TO group. While these were not significant in terms of TO achievement in our analysis, the complexity of the procedure increases the risk of complications, such as anastomotic leakage, bleeding, infection, and pulmonary complications [26]. All incidences of AL reported in our study occurred in patients after THE. These complications can significantly impact the achievement of textbook outcomes during the perioperative period [27]. However, AL did not independently affect the achievement of TO in our study.

Completion of NCT was also shown to significantly improve the chances of achieving TO, possibly due to its effect on tumor response and correlation with overall patient health. Tumor size is a known predictor of surgical outcome, and shrinking the tumor before surgery can increase the likelihood of achieving TO criteria such as R status. While there was no difference in T-stage as objectified by TNM classification, this may not reflect tumor size ranges within a T-stage. Finally, completion of NCT may be a surrogate parameter that reflects the patient’s general condition and health. We saw significantly older patients with a higher CCI Index in the non-TO group. Patients who are able to complete NCT may be in better overall health and have fewer comorbidities that could impact their surgical outcome and recovery. The completion of NCT can also provide valuable information on the tumor’s response to treatment. The difference seen in terms of survival and disease free survival may have been influenced by the increased rate of incomplete NCT in the TO group.

The disease-free survival rate at 1 year was 77.9% (non-TO) and 87.7% (TO), which is in line with other studies [6,23,24]. Several studies in the field of upper GI surgery have also shown improved OS and DFS if TO is achieved [15]. Levy et al. showed that TO patients had a higher 3-year survival rate compared to non-TO patients (75% vs. 55%). This is in line with our results, which showed that the achievement of TO results in an improved OS and DFS [14,15,16]. However, we saw both an increased completion of neoadjuvant chemotherapy in the patients with TO, which may affect improved OS seen in this group. This principal can also be applied when observing disparity in terms of the types of chemotherapy received by both groups; the healthier patients were more likely to receive FLOT, while patients with more comorbidities received FLO. Finally, adjuvant therapy was also completed at a significantly higher rate in the TO group and more commonly consisted of FLOT. While this may also be partly attributed to the distribution of patient demographics, the achievement of TO may also contribute independently to the increased conversion to adjuvant chemotherapy in this group. In such cases, the decision not to pursue adjuvant therapy might not be a deliberate choice but rather a consequence of the deterioration of the patient’s overall health due to surgical complications. As evidenced by the FLOT4 trial, only 46% of patients who have an indication for adjuvant chemotherapy actually complete the treatment. Besides issues such as toxicity, a complication-free surgery within the designated time frame also plays a crucial role. All parameters that result in a hospital stay of more than 30 days automatically render adjuvant chemotherapy impossible [28]. Survival outcomes may have also be influenced by histopathological tumor subtypes. Both GC and GEJ were included due to their comparable prognosis [29,30]. Most tumors were well or moderately differentiated and there was no significant difference seen between the TO groups. However, some additional molecular pathologic data suggest that particular molecular genetic backgrounds within this merged group of tumors may show prognostic variability. Further molecular analysis is not presented in this study and is therefore not accounted for in survival analysis.

We propose that certain criteria should be modified within the TO definition. Peri-operative reinterventions fall under CD 3a and above and are therefore already considered as CD ≥3a. Similarly, unplanned ICU re-admissions are already classified as CD 4, which makes them ineligible for a TO. These criteria provide nuanced information on postoperative complications, relevant for the optimization of patient care. However, we believe that it is unnecessary to evaluate them in terms of TO as they are already accounted for in the CD classification system. The criteria for defining TO should be based on their impact on patient outcomes and feasibility of implementation in routine clinical practice. The use of a simple and standardized definition of TO could facilitate comparisons between studies, hospitals and help identify areas for improvement in surgical practice.

The current study has several limitations that must be considered when interpreting the results. Firstly, the study design is retrospective, which may introduce bias in the data. Secondly, the sample size is small and the study did not account for factors such as ethnicity, socioeconomic status, and comorbidities that could affect the outcomes. Additionally, the lack of clear definitions for TO in gastric surgery limits the comparability of the study with previous research. However, the study may still have an impact in the field by contributing to the development of a standardized TO metric, which could improve surgical quality control and oncological outcomes.

## 5. Conclusions

TO is a quality indicator that is not exclusively limited to reporting morbidity and mortality, but more comprehensively represents a desired postoperative course. We identified minimally invasive surgery as an independent factor positively influencing TO. Furthermore, we showed that TO significantly influences OS and DFS. The implementation of a uniform TO metric would allow for more robust quality control between centers. Therefore, future studies into TO definition are warranted.

## Figures and Tables

**Table 1:** Reasons for failure to achieve Textbook outcome.

Textbook Outcome Metric	Not Achieved *n* = 47
Incomplete Resection	3 (3.1%)
Nodes Sampled ≤ 25	9 (9.1%)
Postoperative severe complications (CD ≥ 3b)	19 (19.2%)
Pulmonary	11(11.11%)
Infectious	8 (8.1%)
Pneumonia	7 (7.1%)
Anastomotic Leak (AL)	6 (6.1%)
Cardiac	5 (5.1%)
Renal	5 (5.1%)
Thromboembolic	4 (4.0%)
Hepatic	1 (1.0%)
Reinterventions	17 (17.2%)
Esophagogastroduodenoscopy (EGD)	10 (10.1%)
Drainage	14 (14.1%)
Other	5 (5.1%)
Unplanned ICU/IMC	14 (14.1%)
Prolonged Hospital stay > 21 Days	15 (15.1%)
Readmission within 30-Days	7 (7.1%)
SSI	3 (3.03%)
GI Bleeding	2 (1.8%)
Pain	2 (1.8%)
Other	1 (1.0%)
Mortality within 30-Days	2 (1.8%)

**Table 2:** Patient characteristics and perioperative outcomes.

Characteristic	Total	Textbook Outcome *n* = 52	Non-Textbook Outcome *n* = 47	*p*-Value
Sex				0.99
Male Female	61 38	32 (61.5%) 20 (38.5%)	29 (61.7%) 18 (38.3%)	
Age (Years)		61.27 (29–90) ^2^	67.04 (33–86) ^2^	**0.03**
BMI (kg/m^2^)		25.06 (+4.18) ^1^	26.34 (5.32) ^1^	0.195
Pre-OP Hemoglobin (g/dL)		11.93 (1.40) ^1^	12.04 (1.65) ^1^	0.74
Pre-OP Anemia (male < 13.5, female < 12 g/dL)	67	38 (74.5%)	29 (63.0%)	0.22
ASA Score				0.19
1 2 3 4	1 40 52 1	1 (2.0%) 25 (50.0%) 23 (46.0%) 1 (2.0%)	0 (0%) 15 (34.1%) 29 (65.9%) 0 (0%)	
CCI tertiles				0.08
≤4 ≥5	45 54	28 (53.8%) 24 (46.2%)	17 (36.2%) 30 (63.8%)	
Cardiovascular Disease	59	28 (53.8%)	31 (66.0%)	0.22
Pulmonary Disease	31	15 (28.8%)	16 (34.0%)	0.58
Renal Disease	4	2 (3.8%)	2 (4.3%)	0.92
Hepatic Disease	1	0 (0%)	1 (2.1%)	0.29
Diabetes Mellitus	16	9 (17.3%)	7 (14.9%)	0.75
Neurological Disease	14	5 (9.6%)	9 (19.1%)	0.17
Prior Abdominal Operation *	33	19 (36.5%)	14 (29.8%)	0.48

*n* (%); ^1^ Mean (SD); ^2^ Mean (Range). ASA—American Society of Anesthesiologists, BMI—Body Mass Index, CCI—Charlson Comorbidity Index, SD—Standard deviation. * Prior abdominal surgery includes all operations for benign conditions, and patients with prior or additional malignancies were excluded.

**Table 3:** Tumor location and histopathological characteristics of patients.

Characteristic	Total	TO *n* = 52	Non-TO *n* = 47	*p*-Value
Tumor Location				0.22
Gastroesophageal Junction (GEJ)	39	17 (32.1%)	22 (46.8%)	
Gastric	60	35 (67.3%)	25 (53.2%)	
Lauren subtype				0.81
Diffuse Intestinal Mixed	30 26 9	17 (47.2%) 13 (36.1%) 6 (16.7%)	13 (48.1%) 11 (40.7%) 3 (11.1%)	
Adenocarcinoma				0.35
G2–G3 Adenocarcinoma * Signet ring cell carcinoma (SRCC) Partial SRCC	57 23 19	27 (51.9%) 15 (28.8%) 10 (19.2%)	30 (63.8%) 8 (17.0%) 9 (19.1%)	
Pre-T Stage				
T1 T2 T3 T4	10 16 43 20	3 (6.3%) 7 (14.6%) 24 (50.0%) 14 (29.2%)	7 (17.1%) 9 (22.0%) 19 (46.3%) 6 (14.6%)	
Pre-N Stage				0.16
N0 N1 N2 N3	31 45 11 2	14 (29.2%) 24 (50.0%) 8 (16.7%) 2 (4.2%)	17 (41.5%) 21 (51.2%) 3 (7.3%) 0 (0%)	
Post-T Stage				0.24
T0 T1 T2 T3 T4	10 24 19 39 6	6 (11.5%) 11 (21.2%) 12 (23.1%) 21 (40.4%) 2 (3.8%)	4 (8.7%) 13 (28.3%) 7 (15.2%) 18 (39.1%) 4 (8.7%)	
Post-N Stage				0.66
N0 N1 N2 N3	62 17 7 12	32 (61.5%) 9 (17.3%) 3 (5.8%) 8 (15.4%)	30 (65.2%) 8 (17.4%) 4 (8.7%) 4 (8.7%)	
Grade (G)				0.74
G2 G3	16 54	5 (15.2%) 28 (84.8%)	11 (29.7%) 26 (70.3%)	
Venous Invasion (V)				0.15
V0 V1	94 4	51 (98.1%) 1 (1.9%)	43 (93.5%) 3 (6.5%)	
Lymphatic vessel Invasion (L)				0.25
L0 L1	73 24	39 (75.0%) 13 (25.0%)	34 (75.6%) 11 (24.4%)	
Perineural invasion (PN)				0.95
PN0 PN1	44 18	22 (68.8%) 10 (31.3%)	22 (73.3%) 8 (26.7%)	

* Comprises tubular, papillary and mucinous adenocarcinoma.

**Table 4:** Multimodal Therapy and Perioperative Characteristics.

Characteristic	Total	Textbook Outcome *n* = 52	Non-Textbook Outcome *n* = 47	*p*-Value
NCT				**0.02**
None received	13	2 (3.8%)	11 (23.4%)	
FLOT	67	41 (78.8%)	26 (55.3%)	
FLO	16	7 (13.5%)	9 (19.1%)	
Other	3	2 (3.8%)	1 (2.1%)	
Discontinued NCT				**0.04**
<4 Cycles	19	6 (11.5%)	13 (27.7%)	
≥4 Cycles	80	46 (88.5%)	34 (72.3%)	
Adjuvant Chemotherapy				0.08
None	17	4 (14.8%)	13 (44.8%)	
FLOT	24	15 (55.6%)	9 (31.0%)	
FLO	8	5 (18.5%)	3 (10.3%)	
Other	7	3 (11.1%)	4 (13.8%)	
Discontinued Adjuvant Chemotherapy				**0.05**
<4 Cycles	28	11 (40.7%)	17 (68.0%)	
≥4 Cycles	24	16 (59.3%)	8 (32.0%)	
Type of resection				0.15
Total	62	36 (69.2%)	26 (55.3%)	
Trans-hiatal Extended Gastrectomy	37	16 (30.8%)	21 (44.7%)	
Anastomosis				0.27
25mm Circular	86	40(85.1%)	46(88.5%)	
29mm Circular	5	4(8.5%)	1(1.9%)	
Linear	8	3(6.5%)	5(9.6%)	
Surgical Approach				**0.02**
Open Laparoscopic	27 72	8 (15.4%) 44 (84.6%)	19 (40.4%) 28 (59.6%)	
Operation Duration (min)		287.62 ^1^	287.87 ^1^	0.99
Days of Stay		12.67 ^1^	21.28 ^1^	**<0.01**

^1^ *n* (%); Mean (SD); NCT-neoadjuvant chemotherapy. Significant values are indicated in bold.

**Table 5:** Logistic Regression Analysis of Factors affecting TO.

	Univariate Analysis		Multivariate Analysis	
	*p*-Value	OR (CI 95%)	*p*-Value	OR (CI 95%)
Age	**0.03**	**6.997 (1.133–43.212)**	0.27	3.626 (0.362–36.240)
Hyperlipidemia	**0.02**	**2.966 (1.171–7.504)**	0.35	1.724 (0.547–5.430)
Tumor Location (AEG)	0.22	0.601 (0.265–1.360)		
ASA	0.07	0.477 (0.212–1.076)		
CCI	0.15	0.530 (0.220–1.279)		
Trans-hiatal Extended Gastrectomy	0.15	0.550 (0.241–1.253)		
Open Surgery	**<0.01**	**3.894 (1.528–10.699)**	**0.01**	**3.715 (1.334–10.351)**
Discontinued NCT	**<0.01**	**7.638 (1.595–36.584)**	**0.02**	**4.278 (1.176–15.553)**

ASA—American Society of Anesthesiologists, CCI—Charlson Comorbidity Index, OR—odds ratio. Significant values are indicated in bold.

**Table 6:** Factors affecting disease free survival after gastrectomy for gastric cancer.

	Overall Survival		Disease Free Survival			
	Univariate Analysis		Univariate Analysis		Multivariate Analysis	
	*p*-Value	OR (CI 95%)	*p*-Value	OR (CI 95%)	*p*-Value	OR (CI 95%)
TO	**0.02**	**0.135 (0.027–0.681)**	**0.04**	**0.448 (0.211–0.954)**	0.069	0.496 (0.232–1.057)
Sex (Male)	0.69	1.326 (0.331–5.309)	0.21	1.634 (0.741–3.600)		
Age	0.76	0.992 (1.044–1.007)	0.09	1.025 (0.996–1.057)		
BMI > 30	0.35	0.409 (0.051–3.275)	0.34	0.639 (0.243–1.680)		
UICC > 2a	0.37	2.407 (0.289–20.005)	0.37	1.536 (0.621–3.805)		
CCI > 4	0.22	3.634 (0.454–29.084)	**0.03**	**2.844 (1.079–7.493)**	**0.035**	**2.605 (0.983–6.905)**
Completion of Neoadjuvant Treatment	0.21	0.409 (0.102–1.640)	0.41	0.672 (0.273–1.653)		
Open Surgery	0.95	1.045 (0.213–5.137)	0.90	1.059 (0.425–2.635)		
ASA > 2	0.45	1.682 (0.419–6.754)	0.70	1.153 (0.551–2.412)		
Tumor Location (AEG)	0.11	2.935 (0.781–11.032)	0.32	1.474 (0.694–3.130)		
Trans-hiatal-Extended Gastrectomy	0.12	2.973 (0.791–11.174)	0.31	1.483 (0.699–3.149)		

Total patients included *n* = 86, non-TO = 34, TO = 52. AEG—Adenocarcinoma of the Esophagogastric junction, ASA—American Society of Anesthesiologists, BMI—Body Mass Index, CCI—Charlson Comorbidity Index, CI—Confidence Interval, OR—odds ratio, SRCC—Signet ring cell carcinoma, UICC—Union Internationale Contre le Cancer. Significant values are indicated in bold.

## Data Availability

Data available from authors upon request.

## References

[1] Morgan E., Arnold M., Camargo M.C., Gini A., Kunzmann A.T., Matsuda T., Meheus F., Verhoeven R.H., Vignat J., Laversanne M. (2022). The current and future incidence and mortality of gastric cancer in 185 countries, 2020–2040: A population-based modelling study. eClinicalMedicine.

[2] Cheng J., Cai M., Shuai X., Gao J., Wang G., Tao K. (2019). Multimodal treatments for resectable gastric cancer: A systematic review and network meta-analysis. Eur. J. Surg. Oncol. EJSO.

[3] Zheng-Yan L., Yong-Liang Z., Feng Q., Yan S., Pei-Wu Y. (2021). Morbidity and short-term surgical outcomes of robotic versus laparo-scopic distal gastrectomy for gastric cancer: A large cohort study. Surg. Endosc..

[4] Lu J., Wu D., Wang H.-G., Zheng C.-H., Li P., Xie J.-W., Wang J.-B., Chen Q.-Y., Cao L.-L., Lin M. (2021). 114O Assessment of robotic versus laparoscopic distal gastrectomy for gastric cancer: A randomized controlled trial. Ann. Oncol..

[5] Wang S., Xu L., Wang Q., Li J., Bai B., Li Z., Wu X., Yu P., Li X., Yin J. (2019). Postoperative complications and prognosis after radical gastrectomy for gastric cancer: A systematic review and meta-analysis of observational studies. World J. Surg. Oncol..

[6] Jin L.X., Sanford D.E., Squires M.H., Moses L.E., Yan Y., Poultsides G.A., Votanopoulos K.I., Weber S.M., Bloomston M., Pawlik T.M. (2016). Interaction of Postoperative Morbidity and Receipt of Adjuvant Therapy on Long-Term Survival After Resection for Gastric Adenocarcinoma: Results from the U.S. Gastric Cancer Collaborative. Ann. Surg. Oncol..

[7] Li S.S., Costantino C.L., Mullen J.T. (2019). Morbidity and Mortality of Total Gastrectomy: A Comprehensive Analysis of 90-Day Outcomes. J. Gastrointest. Surg..

[8] Zhou J., Yu P., Shi Y., Tang B., Hao Y., Zhao Y., Qian F. (2015). Evaluation of Clavien–Dindo classification in patients undergoing total gastrectomy for gastric cancer. Med. Oncol..

[9] Raziee H.R., Cardoso R., Seevaratnam R., Mahar A., Helyer L., Law C., Coburn N. (2012). Systematic review of the predictors of positive margins in gastric cancer surgery and the effect on survival. Gastric Cancer.

[10] Khanjani N., Mirzaei S., Nasrolahi H., Hamedi S.H., Mosalaei A., Omidvari S., Ahmadloo N., Ansari M., Sobhani F., Mohammadianpanah M. (2019). Insufficient lymph node assessment in gastric adenocarcinoma. J. Egypt. Natl. Cancer Inst..

[11] Wang B., Nolan R., Marshall H. (2022). COVID-19 Immunisation, Willingness to Be Vaccinated and Vaccination Strategies to Improve Vaccine Uptake in Australia. Vaccines.

[12] Marano L., Verre L., Carbone L., Poto G.E., Fusario D., Venezia D.F., Calomino N., Kaźmierczak-Siedlecka K., Polom K., Marrelli D. (2023). Current Trends in Volume and Surgical Outcomes in Gastric Cancer. J. Clin. Med..

[13] Busweiler L.A.D., Schouwenburg M.G., Henegouwen M.I.v.B., Kolfschoten N.E., de Jong P.C., Rozema T., Wijnhoven B.P.L., van Hillegersberg R., Wouters M.W.J.M., van Sandick J.W. (2017). Textbook outcome as a composite measure in oesophagogastric cancer surgery. Br. J. Surg..

[14] Kalff M.C., van Berge Henegouwen M.I., Gisbertz S.S. (2021). Textbook outcome for esophageal cancer surgery: An international con-sensus-based update of a quality measure. Dis. Esophagus.

[15] Levy J., Gupta V., Amirazodi E., Allen-Ayodabo C., Jivraj N., Jeong Y., Davis L.E., Mahar A.L., De Mestral C., on behalf of the PRESTO Group (2020). Gastrectomy case volume and textbook outcome: An analysis of the Population Registry of Esophageal and Stomach Tumours of Ontario (PRESTO). Gastric Cancer.

[16] Soh C.H., Hassan S.W.U., Sacre J., Maier A.B. (2020). Morbidity Measures Predicting Mortality in Inpatients: A Systematic Review. J. Am. Med. Dir. Assoc..

[17] Moehler M., Baltin C.T.H., Ebert M., Fischbach W., Gockel I., Grenacher L., Hölscher A.H., Lordick F., Malfertheiner P., Messmann H. (2015). International comparison of the German evidence-based S3-guidelines on the diagnosis and multimodal treatment of early and locally advanced gastric cancer, including adenocarcinoma of the lower esophagus. Gastric Cancer.

[18] Lee S., Lee H., Song J.H., Choi S., Cho M., Son T., Kim H.-I., Hyung W.J. (2020). Intracorporeal esophagojejunostomy using a linear stapler in laparoscopic total gastrectomy: Comparison with circular stapling technique. BMC Surg..

[19] Brierley J.D., Gospodarwicz M.K., Wittekind C., Amin M.B. (2017). TNM Classification of Maligant Tumours.

[20] Clavien P.A., Barkun J., de Oliveira M.L., Vauthey J.N., Dindo D., Schulick R.D., de Santibañes E., Pekolj J., Slankamenac K., Bassi C. (2009). The Clavien-Dindo Classification of Surgical Complications: Five-year experience. Ann. Surg..

[21] Sędłak K., Rawicz-Pruszyński K., Mlak R., Gęca K., Skórzewska M., Pelc Z., Małecka-Massalska T., Polkowski W.P. (2022). Union is strength: Textbook outcome with perioperative chemotherapy compliance decreases the risk of death in advanced gastric cancer patients. Eur. J. Surg. Oncol. EJSO.

[22] Japanese Gastric Cancer Association (2017). Japanese gastric cancer treatment guidelines 2014 (ver. 4). Gastric Cancer.

[23] Chen Q., Ning Z., Liu Z., Zhou Y., He Q., Tian Y., Hao H., Lin W., Jiang L., Zhao G. (2021). Textbook Outcome as a measure of surgical quality assessment and prognosis in gastric neuroendocrine carcinoma: A large multicenter sample analysis. Chin. J. Cancer Res..

[24] Bolger J.C., Al Azzawi M., Whooley J., Bolger E.M., Trench L., Allen J., Kelly M.E., Brosnan C., Arumugasamy M., Robb W.B. (2021). Surgery by a minimally invasive approach is associated with improved textbook outcomes in oesophageal and gastric cancer. Eur. J. Surg. Oncol. EJSO.

[25] Garbarino G.M., Laracca G.G., Lucarini A., Piccolino G., Mercantini P., Costa A., Tonini G., Canali G., Muttillo E.M., Costa G. (2022). Laparoscopic versus Open Surgery for Gastric Cancer in Western Countries: A Systematic Review and Meta-Analysis of Short- and Long-Term Outcomes. J. Clin. Med..

[26] Blank S., Schmidt T., Heger P., Strowitzki M.J., Sisic L., Heger U., Nienhueser H., Haag G.M., Bruckner T., Mihaljevic A.L. (2018). Surgical strategies in true adenocarcinoma of the esophagogastric junction (AEG II): Thoracoabdominal or abdominal approach?. Gastric Cancer.

[27] Fransen L.F.C., Berkelmans G.H.K., Asti E., Henegouwen M.I.v.B., Berlth F., Bonavina L., Brown A., Bruns C., van Daele E., Gisbertz S.S. (2020). The Effect of Postoperative Complications After Minimally Invasive Esophagectomy on Long-term Survival. Ann. Surg..

[28] Al-Batran S.-E., Homann N., Pauligk C., Goetze T.O., Meiler J., Kasper S., Kopp H.-G., Mayer F., Haag G.M., Luley K. (2019). Perioperative chemotherapy with fluorouracil plus leucovorin, oxaliplatin, and docetaxel versus fluorouracil or capecitabine plus cisplatin and epirubicin for locally advanced, resectable gastric or gastro-oesophageal junction adenocarcinoma (FLOT4): A randomised, phase 2/3 trial. Lancet.

[29] Alvarez-Manceñido F., Jimenez-Fonseca P., Carmona-Bayonas A., Arrazubi V., Hernandez R., Cano J.M., Custodio A., Pijaume C.P., Aguado G., Lago N.M. (2021). Is advanced esophageal adenocarcinoma a distinct entity from intestinal subtype gastric cancer? Data from the AGAMENON-SEOM Registry. Gastric Cancer.

[30] Davis J.A., Cui Z.L., Ghias M., Li X., Goodloe R., Wang C., Liepa A.M., Hess L.M. (2022). Treatment heterogeneity and overall survival in patients with advanced/metastatic gastric or gastroesophageal junction adenocarcinoma in the United States. J. Gastrointest. Oncol..

